# 2,4-Bis(4-chloro­benzo­yl)-1-(4-chloro­phen­yl)-3,5-di-2-thienylcyclo­hexa­nol methanol hemisolvate

**DOI:** 10.1107/S1600536808008428

**Published:** 2008-04-02

**Authors:** Xiao-Fang Wang, Xian-Qiang Huang

**Affiliations:** a College of Life Sciences and Chemistry, Tianshui Normal University, Tianshui 741000, People’s Republic of China; b College of Chemistry and Chemical Engineering, Liaocheng University, Shandong 252059, People’s Republic of China

## Abstract

The title compound, C_34_H_25_Cl_3_O_3_S_2_·0.5CH_3_OH, was synthesized by the reaction of thio­phene-2-carbaldehyde with acetophenone and NaOH under solvent-free conditions, using tetra­butylammonium bromide as a phase-transfer catalyst. The central six-membered ring adopts a chair conformation with the bulky thio­phene, 4-chloro­phenyl and 4-chloro­benzoyl substituents in equatorial positions. The hydroxyl group is in an axial position and forms an intra­molecular O—H⋯O hydrogen bond to the carbonyl group of an adjacent 4-chloro­benzoyl substituent. The methanol solvent mol­ecules are disordered equally over two positions within one-dimensional channels, with site occupancy factors of 0.25.

## Related literature

For related structures, see: Luo & Shan (2006 ▶); Huang & Wang (2007 ▶).
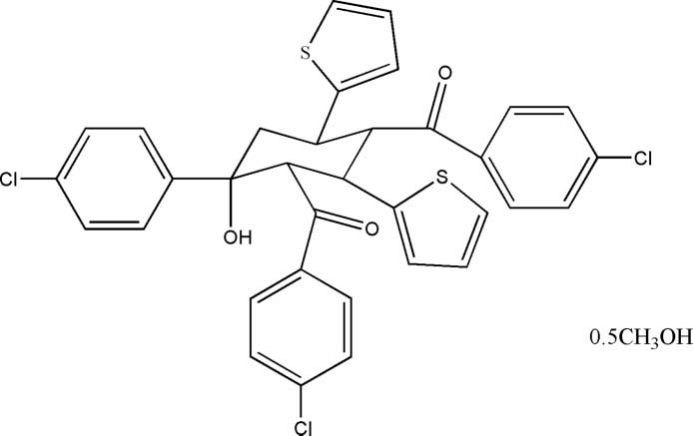


## Experimental

### 

#### Crystal data

C_34_H_25_Cl_3_O_3_S_2_·0.5CH_4_O
*M* *_r_* = 668.03
Orthorhombic, 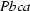

*a* = 22.5660 (16) Å
*b* = 12.1356 (12) Å
*c* = 26.030 (2) Å
*V* = 7128.4 (11) Å^3^
*Z* = 8
Mo *K*α radiation
μ = 0.41 mm^−1^
*T* = 298 (2) K
0.67 × 0.16 × 0.13 mm

#### Data collection

Bruker SMART CCD diffractometer
Absorption correction: multi-scan (*SADABS*; Sheldrick, 2003 ▶) *T*_min_ = 0.772, *T*_max_ = 0.949
27584 measured reflections
4502 independent reflections
2357 reflections with *I* > 2σ(*I*)
*R*_int_ = 0.096
θ_max_ = 22.5°

#### Refinement

*R*[*F* ^2^ > 2σ(*F* ^2^)] = 0.080
*wR*(*F* ^2^) = 0.290
*S* = 1.09
4502 reflections
415 parameters
50 restraints
H-atom parameters constrained
Δρ_max_ = 0.64 e Å^−3^
Δρ_min_ = −0.40 e Å^−3^

### 

Data collection: *SMART* (Bruker, 2001 ▶); cell refinement: *SAINT* (Bruker, 2001 ▶); data reduction: *SAINT*; program(s) used to solve structure: *SHELXS97* (Sheldrick, 2008 ▶); program(s) used to refine structure: *SHELXL97* (Sheldrick, 2008 ▶)); molecular graphics: *SHELXTL* (Sheldrick, 2008 ▶); software used to prepare material for publication: *SHELXTL*.

## Supplementary Material

Crystal structure: contains datablocks I, global. DOI: 10.1107/S1600536808008428/bi2281sup1.cif

Structure factors: contains datablocks I. DOI: 10.1107/S1600536808008428/bi2281Isup2.hkl

Additional supplementary materials:  crystallographic information; 3D view; checkCIF report

## Figures and Tables

**Table 1:** Hydrogen-bond geometry (Å, °)

*D*—H⋯*A*	*D*—H	H⋯*A*	*D*⋯*A*	*D*—H⋯*A*
O1—H1⋯O2	0.82	2.19	2.772 (7)	128

**Fig. 1.**
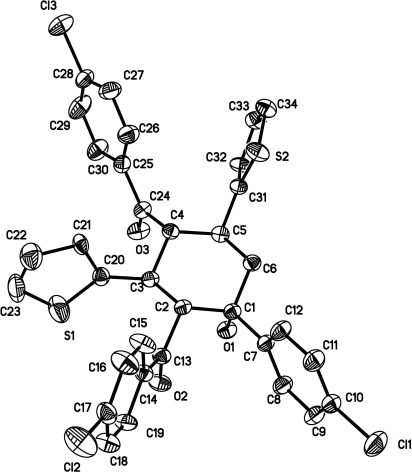
Molecular stucture showing displacement ellipsoids at 20% probability. H atoms and the disordered methanol molecule is omitted.

## supplementary crystallographic information

### Comment

The title compound (Fig. 1) was synthesized by condensation and Michael addition of thiophene-2-carbaldehyde with 4-chloroacetophenone under solvent-free conditions, using tetrabutyl ammonium bromide as a phase-transfer catalyst. The bond lengths and angles are comparable to those observed in the related compounds 2,4-dibenzoyl-3,5-bis(4-methoxylphenyl)-1-phenylcyclohexanol (Luo & Shan, 2006) and 2,4-dibenzoyl-3,5-bis(2-thienyl)-1-phenylcyclohexanol (Huang & Wang, 2007). The hydroxyl group in the axial position forms an intramolecular O—H···O hydrogen bond to the carbonyl group of an adjacent *para*-chlorobenzoyl substituent (Table 1). The methanol solvent molecules lie within channels running along the crystallogaphic *b* axis, and are modelled as disordered along those channels.

### Experimental

4-Chloroacetophenone (6.25 mmol), freshly distilled thiophene-2-carbaldehyde (3.125 mmol), NaOH (6.25 mmol) and tetrabutyl ammonium bromide (1 mmol) were mixed with a glass paddle in an open flask. The resulting mixture was washed several times with water to remove NaOH and recrystallized from methanol to give the title compound as a crystalline solid. Elemental analysis calculated: C 62.03, H 4.07%; found: C 62.08, H 4.02%.

### Refinement

H atoms were positioned geometrically and refined using a riding model with C—H = 0.93–0.98 Å and *U*_iso_(H) = 1.2 or 1.5*U*_eq_(C). The H atoms of hydroxyl groups were placed in idealized positions with O—H = 0.82 Å and refined as riding with *U*_iso_(H) = 1.5*U*_eq_(O). The crystal diffracted relatively weakly and the data are therefore limited to θ_max_ = 22.5°, with *ca* 50% data observed at the 2σ(I) level. The resulting structure is therefore of relatively low precision. The methanol solvent molecule was refined as disordered over two orientations, each with 25% site occupancy to give reasonable displacement parameters. The C—O bonds were restrained to 1.45 (2) Å and the anisotropic displacement parameters of all atoms were restrained to have approximately equal components.

### Crystal data

**Table:**

C_34_H_25_Cl_3_O_3_S_2_·0.5CH_4_O	*F*_000_ = 2760
*M**_r_* = 668.03	*D*_x_ = 1.245 Mg m^−^^3^
Orthorhombic, *P**b**c**a*	Mo *K*α radiation λ = 0.71073 Å
Hall symbol: -P 2ac 2ab	Cell parameters from 3877 reflections
*a* = 22.5660 (16) Å	θ = 2.4–19.6º
*b* = 12.1356 (12) Å	µ = 0.41 mm^−^^1^
*c* = 26.030 (2) Å	*T* = 298 (2) K
*V* = 7128.4 (11) Å^3^	Block, colourless
*Z* = 8	0.67 × 0.16 × 0.13 mm

### Data collection

**Table:**

Bruker SMART CCD diffractometer	4502 independent reflections
Radiation source: fine-focus sealed tube	2357 reflections with *I* > 2σ(*I*)
Monochromator: graphite	*R*_int_ = 0.096
*T* = 298(2) K	θ_max_ = 22.5º
φ and ω scans	θ_min_ = 2.1º
Absorption correction: multi-scan(SADABS; Sheldrick, 2003)	*h* = −24→21
*T*_min_ = 0.772, *T*_max_ = 0.949	*k* = −13→12
27584 measured reflections	*l* = −28→27

### Refinement

**Table:**

Refinement on *F*^2^	Secondary atom site location: difference Fourier map
Least-squares matrix: full	Hydrogen site location: inferred from neighbouring sites
*R*[*F*^2^ > 2σ(*F*^2^)] = 0.080	H-atom parameters constrained
*wR*(*F*^2^) = 0.290	*w* = 1/[σ^2^(*F*_o_^2^) + (0.1474*P*)^2^ + 3.8778*P*] where *P* = (*F*_o_^2^ + 2*F*_c_^2^)/3
*S* = 1.09	(Δ/σ)_max_ = 0.004
4502 reflections	Δρ_max_ = 0.64 e Å^−^^3^
415 parameters	Δρ_min_ = −0.40 e Å^−^^3^
50 restraints	Extinction correction: none
Primary atom site location: structure-invariant direct methods	

### Special details

**Table:**

Geometry. All e.s.d.'s (except the e.s.d. in the dihedral angle between two l.s. planes) are estimated using the full covariance matrix. The cell e.s.d.'s are taken into account individually in the estimation of e.s.d.'s in distances, angles and torsion angles; correlations between e.s.d.'s in cell parameters are only used when they are defined by crystal symmetry. An approximate (isotropic) treatment of cell e.s.d.'s is used for estimating e.s.d.'s involving l.s. planes.
Refinement. Refinement of *F*^2^ against ALL reflections. The weighted *R*-factor *wR* and goodness of fit *S* are based on *F*^2^, conventional *R*-factors *R* are based on *F*, with *F* set to zero for negative *F*^2^. The threshold expression of *F*^2^ > σ(*F*^2^) is used only for calculating *R*-factors(gt) *etc*. and is not relevant to the choice of reflections for refinement. *R*-factors based on *F*^2^ are statistically about twice as large as those based on *F*, and *R*- factors based on ALL data will be even larger.

### Fractional atomic coordinates and isotropic or equivalent isotropic displacement parameters (Å^2^)

**Table:**

	*x*	*y*	*z*	*U*_iso_*/*U*_eq_	Occ. (<1)
Cl1	0.95396 (12)	0.3492 (3)	−0.16180 (9)	0.1142 (10)	
Cl2	1.09441 (11)	0.3656 (2)	0.12369 (13)	0.1225 (11)	
Cl3	0.53719 (14)	0.0925 (3)	0.28831 (11)	0.1374 (13)	
O1	0.8096 (2)	−0.0250 (4)	−0.02208 (17)	0.0638 (13)	
H1	0.8436	−0.0488	−0.0231	0.096*	
O2	0.9156 (2)	−0.0200 (4)	0.0317 (2)	0.0713 (14)	
O3	0.7060 (2)	−0.1231 (5)	0.1052 (2)	0.0798 (16)	
O4	0.769 (2)	0.641 (7)	0.351 (3)	0.26 (2)	0.25
H4	0.7863	0.6794	0.3305	0.395*	0.25
O5	0.747 (2)	0.302 (6)	0.304 (3)	0.27 (2)	0.25
H5	0.7735	0.2800	0.2856	0.401*	0.25
S1	0.86979 (15)	−0.0641 (3)	0.16254 (12)	0.1253 (11)	
S2	0.62891 (9)	0.23441 (18)	0.03888 (10)	0.0801 (7)	
C1	0.8106 (3)	0.0886 (5)	−0.0078 (2)	0.0512 (17)	
C2	0.8364 (3)	0.1015 (5)	0.0474 (2)	0.0486 (16)	
H2	0.8321	0.1784	0.0583	0.058*	
C3	0.8012 (3)	0.0259 (6)	0.0853 (3)	0.0547 (18)	
H3	0.8045	−0.0502	0.0731	0.066*	
C4	0.7355 (3)	0.0580 (6)	0.0849 (2)	0.0506 (17)	
H4A	0.7308	0.1311	0.1004	0.061*	
C5	0.7092 (3)	0.0585 (6)	0.0302 (3)	0.0531 (17)	
H5A	0.7086	−0.0177	0.0177	0.064*	
C6	0.7465 (3)	0.1268 (6)	−0.0074 (3)	0.0565 (18)	
H6A	0.7301	0.1202	−0.0417	0.068*	
H6B	0.7449	0.2039	0.0024	0.068*	
C7	0.8479 (3)	0.1562 (6)	−0.0462 (2)	0.0551 (18)	
C8	0.8786 (3)	0.1061 (7)	−0.0853 (3)	0.073 (2)	
H8	0.8770	0.0298	−0.0884	0.088*	
C9	0.9118 (4)	0.1661 (9)	−0.1201 (3)	0.084 (3)	
H9	0.9327	0.1301	−0.1459	0.101*	
C10	0.9137 (3)	0.2763 (8)	−0.1166 (3)	0.071 (2)	
C11	0.8847 (4)	0.3293 (8)	−0.0774 (3)	0.080 (2)	
H11	0.8871	0.4055	−0.0742	0.096*	
C12	0.8519 (4)	0.2680 (7)	−0.0428 (3)	0.069 (2)	
H12	0.8321	0.3041	−0.0164	0.083*	
C13	0.9012 (3)	0.0708 (6)	0.0487 (3)	0.0547 (18)	
C14	0.9471 (3)	0.1467 (6)	0.0680 (3)	0.0529 (17)	
C15	0.9353 (3)	0.2480 (6)	0.0891 (3)	0.078 (2)	
H15	0.8962	0.2710	0.0924	0.093*	
C16	0.9803 (4)	0.3164 (7)	0.1056 (4)	0.090 (3)	
H16	0.9717	0.3859	0.1187	0.108*	
C17	1.0372 (3)	0.2813 (8)	0.1023 (3)	0.072 (2)	
C18	1.0507 (4)	0.1813 (8)	0.0824 (3)	0.079 (2)	
H18	1.0900	0.1588	0.0802	0.095*	
C19	1.0067 (3)	0.1142 (7)	0.0655 (3)	0.070 (2)	
H19	1.0162	0.0454	0.0520	0.084*	
C20	0.8272 (3)	0.0314 (6)	0.1381 (3)	0.0601 (19)	
C21	0.8207 (3)	0.1219 (6)	0.1774 (2)	0.0587 (18)	
H21	0.7972	0.1844	0.1740	0.070*	
C22	0.8565 (5)	0.0956 (10)	0.2202 (4)	0.112 (3)	
H22	0.8614	0.1430	0.2479	0.135*	
C23	0.8815 (5)	0.0013 (12)	0.2177 (4)	0.120 (4)	
H23	0.9041	−0.0280	0.2442	0.144*	
C24	0.7011 (3)	−0.0268 (7)	0.1167 (3)	0.0565 (18)	
C25	0.6618 (3)	0.0078 (7)	0.1602 (3)	0.0610 (19)	
C26	0.6492 (4)	0.1131 (8)	0.1721 (3)	0.083 (2)	
H26	0.6673	0.1691	0.1534	0.100*	
C27	0.6109 (4)	0.1411 (9)	0.2108 (4)	0.096 (3)	
H27	0.6027	0.2146	0.2180	0.115*	
C28	0.5848 (4)	0.0586 (10)	0.2387 (3)	0.085 (3)	
C29	0.5948 (5)	−0.0444 (10)	0.2273 (4)	0.109 (3)	
H29	0.5755	−0.0999	0.2454	0.131*	
C30	0.6346 (4)	−0.0719 (8)	0.1880 (3)	0.090 (3)	
H30	0.6424	−0.1456	0.1809	0.108*	
C31	0.6455 (3)	0.0992 (6)	0.0322 (3)	0.0537 (17)	
C32	0.5939 (3)	0.0334 (6)	0.0320 (3)	0.0607 (19)	
H32	0.5929	−0.0427	0.0280	0.073*	
C33	0.5433 (3)	0.1036 (8)	0.0389 (3)	0.080 (2)	
H33	0.5048	0.0766	0.0402	0.095*	
C34	0.5559 (3)	0.2092 (8)	0.0432 (3)	0.078 (2)	
H34	0.5274	0.2637	0.0481	0.094*	
C35	0.756 (4)	0.534 (7)	0.328 (4)	0.26 (2)	0.25
H35A	0.7855	0.5181	0.3027	0.394*	0.25
H35B	0.7177	0.5359	0.3126	0.394*	0.25
H35C	0.7570	0.4783	0.3544	0.394*	0.25
C36	0.752 (3)	0.420 (7)	0.311 (4)	0.26 (2)	0.25
H36A	0.7377	0.4577	0.2814	0.394*	0.25
H36B	0.7931	0.4391	0.3168	0.394*	0.25
H36C	0.7294	0.4426	0.3408	0.394*	0.25

### Atomic displacement parameters (Å^2^)

**Table:**

	*U*^11^	*U*^22^	*U*^33^	*U*^12^	*U*^13^	*U*^23^
Cl1	0.1111 (19)	0.148 (3)	0.0834 (16)	−0.0190 (16)	0.0260 (13)	0.0327 (16)
Cl2	0.0846 (17)	0.096 (2)	0.186 (3)	−0.0247 (14)	−0.0361 (17)	−0.0093 (18)
Cl3	0.131 (2)	0.192 (3)	0.0885 (19)	0.017 (2)	0.0507 (16)	0.0001 (19)
O1	0.074 (3)	0.047 (3)	0.071 (3)	0.001 (2)	0.006 (2)	−0.013 (2)
O2	0.064 (3)	0.053 (3)	0.097 (4)	0.008 (3)	0.006 (3)	−0.017 (3)
O3	0.085 (4)	0.056 (4)	0.099 (4)	−0.012 (3)	0.028 (3)	−0.003 (3)
O4	0.14 (3)	0.34 (7)	0.31 (5)	0.05 (4)	−0.03 (2)	0.05 (5)
O5	0.15 (2)	0.34 (7)	0.31 (5)	0.05 (4)	−0.02 (2)	0.04 (5)
S1	0.145 (3)	0.110 (2)	0.121 (2)	0.0275 (19)	−0.0365 (18)	0.0112 (18)
S2	0.0612 (12)	0.0590 (14)	0.1201 (18)	0.0011 (10)	−0.0021 (11)	−0.0019 (12)
C1	0.055 (4)	0.043 (4)	0.056 (4)	0.003 (3)	0.007 (3)	−0.003 (3)
C2	0.048 (4)	0.044 (4)	0.055 (4)	−0.001 (3)	0.010 (3)	0.000 (3)
C3	0.054 (4)	0.053 (4)	0.057 (4)	0.002 (3)	0.008 (3)	−0.001 (3)
C4	0.045 (4)	0.049 (4)	0.058 (4)	−0.004 (3)	0.003 (3)	0.001 (3)
C5	0.049 (4)	0.044 (4)	0.066 (4)	−0.006 (3)	0.000 (3)	−0.007 (3)
C6	0.049 (4)	0.060 (5)	0.060 (4)	−0.006 (3)	0.004 (3)	0.000 (4)
C7	0.053 (4)	0.065 (5)	0.048 (4)	−0.003 (3)	0.004 (3)	0.002 (4)
C8	0.084 (5)	0.073 (6)	0.062 (5)	0.009 (4)	0.021 (4)	0.002 (4)
C9	0.089 (6)	0.100 (8)	0.063 (5)	0.014 (5)	0.025 (4)	0.002 (5)
C10	0.060 (5)	0.090 (7)	0.061 (5)	−0.002 (5)	0.008 (4)	0.010 (5)
C11	0.084 (5)	0.080 (6)	0.076 (6)	−0.011 (5)	0.013 (5)	0.011 (5)
C12	0.088 (5)	0.055 (5)	0.063 (5)	−0.007 (4)	0.021 (4)	0.001 (4)
C13	0.057 (4)	0.047 (5)	0.060 (4)	0.008 (4)	0.009 (3)	0.001 (4)
C14	0.046 (4)	0.053 (5)	0.059 (4)	−0.002 (3)	0.001 (3)	0.002 (3)
C15	0.052 (4)	0.056 (5)	0.125 (7)	0.006 (4)	−0.010 (4)	−0.018 (5)
C16	0.082 (6)	0.054 (5)	0.136 (8)	0.010 (5)	−0.018 (5)	−0.021 (5)
C17	0.053 (5)	0.084 (7)	0.079 (5)	−0.009 (4)	−0.013 (4)	0.015 (5)
C18	0.057 (5)	0.080 (6)	0.101 (6)	0.005 (5)	0.002 (4)	−0.007 (5)
C19	0.048 (4)	0.078 (6)	0.084 (6)	0.009 (4)	0.006 (4)	−0.017 (4)
C20	0.060 (4)	0.060 (5)	0.060 (4)	−0.006 (4)	−0.002 (3)	0.009 (4)
C21	0.072 (4)	0.051 (4)	0.053 (4)	0.006 (3)	−0.028 (3)	−0.013 (3)
C22	0.128 (6)	0.113 (6)	0.097 (5)	−0.001 (5)	−0.023 (5)	−0.013 (5)
C23	0.118 (8)	0.160 (12)	0.082 (7)	−0.006 (8)	−0.037 (6)	0.036 (7)
C24	0.065 (5)	0.047 (5)	0.057 (4)	−0.009 (4)	0.005 (3)	−0.003 (4)
C25	0.051 (4)	0.073 (6)	0.059 (4)	−0.012 (4)	0.001 (3)	0.006 (4)
C26	0.091 (6)	0.073 (7)	0.085 (6)	0.006 (5)	0.024 (5)	0.008 (5)
C27	0.109 (7)	0.094 (7)	0.085 (6)	0.028 (6)	0.033 (6)	0.005 (6)
C28	0.077 (6)	0.126 (9)	0.052 (5)	0.007 (6)	0.010 (4)	−0.007 (6)
C29	0.156 (10)	0.093 (8)	0.078 (6)	−0.028 (7)	0.036 (7)	0.000 (6)
C30	0.116 (7)	0.071 (6)	0.082 (6)	−0.017 (5)	0.021 (5)	0.005 (5)
C31	0.048 (4)	0.052 (4)	0.061 (4)	−0.002 (3)	0.000 (3)	−0.004 (3)
C32	0.033 (4)	0.059 (5)	0.090 (5)	−0.011 (3)	0.006 (3)	−0.013 (4)
C33	0.041 (4)	0.104 (8)	0.094 (6)	−0.014 (4)	−0.002 (4)	−0.008 (5)
C34	0.060 (5)	0.077 (7)	0.097 (6)	0.002 (4)	−0.002 (4)	0.002 (5)
C35	0.15 (2)	0.34 (7)	0.31 (5)	0.06 (4)	−0.04 (2)	0.05 (5)
C36	0.14 (2)	0.34 (7)	0.31 (5)	0.06 (4)	−0.04 (2)	0.05 (5)

### Geometric parameters (Å, °)

**Table:**

Cl1—C10	1.729 (8)	C12—H12	0.930
Cl2—C17	1.739 (8)	C13—C14	1.475 (10)
Cl3—C28	1.730 (9)	C14—C15	1.374 (10)
O1—C1	1.428 (7)	C14—C19	1.402 (9)
O1—H1	0.820	C15—C16	1.380 (11)
O2—C13	1.230 (8)	C15—H15	0.930
O3—C24	1.211 (8)	C16—C17	1.355 (11)
O4—C35	1.45 (2)	C16—H16	0.930
O4—H4	0.820	C17—C18	1.354 (11)
O5—C36	1.45 (2)	C18—C19	1.358 (11)
O5—H5	0.820	C18—H18	0.930
S1—C20	1.633 (8)	C19—H19	0.930
S1—C23	1.661 (12)	C20—C21	1.508 (10)
S2—C34	1.681 (8)	C21—C22	1.411 (13)
S2—C31	1.692 (7)	C21—H21	0.930
C1—C6	1.519 (9)	C22—C23	1.279 (15)
C1—C7	1.543 (9)	C22—H22	0.930
C1—C2	1.560 (9)	C23—H23	0.930
C2—C13	1.509 (9)	C24—C25	1.498 (10)
C2—C3	1.564 (9)	C25—C26	1.346 (11)
C2—H2	0.980	C25—C30	1.355 (11)
C3—C20	1.497 (10)	C26—C27	1.371 (11)
C3—C4	1.533 (9)	C26—H26	0.930
C3—H3	0.980	C27—C28	1.369 (13)
C4—C24	1.531 (9)	C27—H27	0.930
C4—C5	1.544 (9)	C28—C29	1.304 (13)
C4—H4A	0.980	C29—C30	1.400 (13)
C5—C31	1.520 (9)	C29—H29	0.930
C5—C6	1.535 (9)	C30—H30	0.930
C5—H5A	0.980	C31—C32	1.411 (9)
C6—H6A	0.970	C32—C33	1.436 (11)
C6—H6B	0.970	C32—H32	0.930
C7—C12	1.363 (10)	C33—C34	1.317 (11)
C7—C8	1.372 (10)	C33—H33	0.930
C8—C9	1.383 (11)	C34—H34	0.930
C8—H8	0.930	C35—H35A	0.960
C9—C10	1.341 (12)	C35—H35B	0.960
C9—H9	0.930	C35—H35C	0.960
C10—C11	1.372 (11)	C36—H36A	0.960
C11—C12	1.383 (10)	C36—H36B	0.960
C11—H11	0.930	C36—H36C	0.960
			
C1—O1—H1	109.5	C17—C16—H16	120.4
C35—O4—H4	109.5	C15—C16—H16	120.4
C36—O5—H5	109.5	C18—C17—C16	121.3 (7)
C20—S1—C23	95.2 (5)	C18—C17—Cl2	118.8 (6)
C34—S2—C31	92.7 (4)	C16—C17—Cl2	119.9 (7)
O1—C1—C6	106.3 (5)	C17—C18—C19	119.8 (7)
O1—C1—C7	110.7 (5)	C17—C18—H18	120.1
C6—C1—C7	111.2 (5)	C19—C18—H18	120.1
O1—C1—C2	110.0 (5)	C18—C19—C14	121.2 (8)
C6—C1—C2	108.6 (5)	C18—C19—H19	119.4
C7—C1—C2	109.9 (5)	C14—C19—H19	119.4
C13—C2—C1	110.9 (5)	C3—C20—C21	128.1 (6)
C13—C2—C3	109.5 (5)	C3—C20—S1	123.8 (6)
C1—C2—C3	109.4 (5)	C21—C20—S1	108.1 (5)
C13—C2—H2	109.0	C22—C21—C20	108.3 (7)
C1—C2—H2	109.0	C22—C21—H21	125.8
C3—C2—H2	109.0	C20—C21—H21	125.8
C20—C3—C4	111.9 (5)	C23—C22—C21	114.5 (10)
C20—C3—C2	110.8 (5)	C23—C22—H22	122.7
C4—C3—C2	109.8 (5)	C21—C22—H22	122.7
C20—C3—H3	108.1	C22—C23—S1	113.7 (8)
C4—C3—H3	108.1	C22—C23—H23	123.2
C2—C3—H3	108.1	S1—C23—H23	123.2
C24—C4—C3	108.4 (6)	O3—C24—C25	120.6 (6)
C24—C4—C5	107.8 (5)	O3—C24—C4	118.0 (6)
C3—C4—C5	112.3 (5)	C25—C24—C4	121.3 (7)
C24—C4—H4A	109.4	C26—C25—C30	117.3 (8)
C3—C4—H4A	109.4	C26—C25—C24	124.5 (7)
C5—C4—H4A	109.4	C30—C25—C24	118.2 (8)
C31—C5—C6	111.5 (6)	C25—C26—C27	122.6 (8)
C31—C5—C4	109.5 (5)	C25—C26—H26	118.7
C6—C5—C4	112.3 (5)	C27—C26—H26	118.7
C31—C5—H5A	107.8	C28—C27—C26	118.6 (9)
C6—C5—H5A	107.8	C28—C27—H27	120.7
C4—C5—H5A	107.8	C26—C27—H27	120.7
C1—C6—C5	111.3 (6)	C29—C28—C27	120.4 (8)
C1—C6—H6A	109.4	C29—C28—Cl3	120.4 (9)
C5—C6—H6A	109.4	C27—C28—Cl3	119.2 (9)
C1—C6—H6B	109.4	C28—C29—C30	120.4 (9)
C5—C6—H6B	109.4	C28—C29—H29	119.8
H6A—C6—H6B	108.0	C30—C29—H29	119.8
C12—C7—C8	117.2 (7)	C25—C30—C29	120.7 (9)
C12—C7—C1	121.5 (6)	C25—C30—H30	119.6
C8—C7—C1	121.3 (7)	C29—C30—H30	119.6
C7—C8—C9	121.7 (8)	C32—C31—C5	126.6 (6)
C7—C8—H8	119.2	C32—C31—S2	111.5 (5)
C9—C8—H8	119.2	C5—C31—S2	121.8 (5)
C10—C9—C8	119.9 (8)	C31—C32—C33	108.7 (7)
C10—C9—H9	120.0	C31—C32—H32	125.7
C8—C9—H9	120.0	C33—C32—H32	125.7
C9—C10—C11	120.1 (7)	C34—C33—C32	114.7 (7)
C9—C10—Cl1	118.8 (7)	C34—C33—H33	122.7
C11—C10—Cl1	121.1 (7)	C32—C33—H33	122.7
C10—C11—C12	119.2 (8)	C33—C34—S2	112.4 (6)
C10—C11—H11	120.4	C33—C34—H34	123.8
C12—C11—H11	120.4	S2—C34—H34	123.8
C7—C12—C11	121.9 (7)	O4—C35—H35A	109.5
C7—C12—H12	119.1	O4—C35—H35B	109.5
C11—C12—H12	119.1	H35A—C35—H35B	109.5
O2—C13—C14	119.8 (6)	O4—C35—H35C	109.5
O2—C13—C2	117.9 (6)	H35A—C35—H35C	109.5
C14—C13—C2	122.2 (6)	H35B—C35—H35C	109.5
C15—C14—C19	117.2 (7)	O5—C36—H36A	109.5
C15—C14—C13	124.0 (6)	O5—C36—H36B	109.5
C19—C14—C13	118.8 (7)	H36A—C36—H36B	109.5
C14—C15—C16	121.3 (7)	O5—C36—H36C	109.5
C14—C15—H15	119.4	H36A—C36—H36C	109.5
C16—C15—H15	119.4	H36B—C36—H36C	109.5
C17—C16—C15	119.2 (8)		

### Hydrogen-bond geometry (Å, °)

**Table:**

*D*—H···*A*	*D*—H	H···*A*	*D*···*A*	*D*—H···*A*
O1—H1···O2	0.82	2.19	2.772 (7)	128
